# Centering disability visibility in reproductive health care: Dismantling barriers to achieve reproductive equity

**DOI:** 10.1177/17455057231197166

**Published:** 2023-09-07

**Authors:** Jordan Fletcher, Halina Yee, Bonnie Ong, Rosemary Claire Roden

**Affiliations:** 1 Department of Obstetrics and Gynecology, The Pennsylvania State University, Penn State College of Medicine, Hershey, PA, USA; 2 Department of Medical Anthropology, University College London, London, UK; 3 Division of Adolescent Medicine, Department of Pediatrics, The Pennsylvania State University, Penn State College of Medicine, Hershey, PA, USA

**Keywords:** abortion, contraception, disability, justice, reproductive health care

## Abstract

Access to comprehensive and culturally competent reproductive health care is essential for individuals and communities to realize and achieve health and well-being, as one prefers. The disability community represents a diverse group of individuals with a wide spectrum of functional, physical, sensory, and/or neurodivergent abilities. Existing barriers to reproductive health care are a consequence of environmental and attitudinal barriers, not from the disabilities themselves. People with disabilities are also not frequently centered or included in discussions surrounding reproductive rights. This article reviews the intersection of the Disability Justice Movement and the history of discrimination in the United States against people with disabilities with a particular focus on reproductive oppression. We discuss the mechanisms of inequity and barriers to health care, including financial barriers, inaccessible medical facilities, provider discrimination and competency, and guardianship; as well as the importance of open access to contraception, menstrual health, and abortion for people with disabilities. Finally, we explore the intersection of the Disability Justice Movement and the Reproductive Justice Movement to better promote reproductive autonomy.

## Plain Language Summary

“Improving the inclusivity of reproductive care—shining the spotlight on individuals with disabilities”

In June 2022, the US Supreme Court reversed the constitutional right to abortion in the *Dobbs vs. Jackson Women’s Health Organization* ruling. This has sparked outcries from the Reproductive Justice Movement regarding the critical importance to make decisions about whether and when to get pregnant, whether and when to prevent pregnancy, and how to manage unwanted pregnancies. Yet, individuals with disabilities are frequently not centered or included in discussions surrounding reproductive rights. This is especially troubling due to a long-standing history of marginalization in this population. In this article, we review how we can approach health care from an inclusivity perspective to dismantle implicit biases, confront systemic barriers, and embrace the diversity of those with disabilities. We illuminate the US history of discrimination and stigmatization against people with disabilities specific to contraception and abortion, and discuss barriers to equitable reproductive health care. Finally, we offer guidance on improving access, quality, delivery of inclusive health care, and centering the patient experience.

## Introduction

Access to comprehensive and culturally competent reproductive health care is essential for individuals and communities to realize and achieve health and well-being, as one prefers. The term “disability” encompasses a wide spectrum of functional, physical, sensory, and/or neurodivergent abilities. It includes a diverse population who have the same inherent rights as their counterparts to bodily autonomy, self-determination, parenthood, and accessible, affordable health care, including contraception and abortion, that is free from discrimination and stigmatization.^1–4^ Significant obstacles to accessing this care persist due to societal and structural barriers that hinder their ability to obtain comprehensive and culturally competent reproductive health care, not from the disabilities themselves.^1,5^

The Disability Rights Movement has long recognized the structural barriers that impair these communities’ acquisition of comprehensive health care. It originated in the United States in an effort to expose and oppose the barriers to everyday living imposed by societal constructs on the disability community.^6^ The passage of the Americans with Disabilities Act (ADA) in 1990 fought to secure comprehensive protections for the basic civil rights of people with disabilities, prohibit discrimination, and mandate accessibility.^6^ Self-advocacy groups such as the Disability Rights Education and Defense Fund, Americans Disabled Attendant Programs Today, and the Center for Independent Living have also helped shape national conversation around disability. Despite its passage, many structural barriers to health care persist,^7^ including barriers to equitable housing,^8^ equitable work opportunities,^9^ and comprehensive sexual health education.^10^

Activists like Judy Heumann and Ed Roberts fought for improved representation for all people with disabilities^7^ and worked to expose the many persisting societal barriers that occlude their access to health care services. Historical discrimination and coercive reproductive practice, facility inaccessibility, and lack of culturally competent care are several barriers to reproductive autonomy imposed on the disability community by those who are not a part of it.^3^ Social and economic marginalization and lack of representation from the disability community have also hindered the progress of inclusive reproductive policies and health care provision.^3^

The recent decision by the US Supreme Court^11^ in *Dobbs v. Jackson Women’s Health Organization* has continued to erode reproductive access for all, in particular, multi-marginalized and minoritized communities including Queer communities, communities of color, and individuals with disabilities.^3^ In the United States in 2022 alone, 41 states introduced more than 500 abortion restrictions.^3^ These attacks on reproductive health care have a disproportionate impact on already marginalized individuals and communities, particularly the disability community.^3^ It is critical that the accessibility and affordability of full-scope contraception are prioritized for members of the disability community.^12^

Thus, this review aims to align the vision of trailblazers of the Disability Rights Movement with that of the Reproductive Justice Movement to center and amplify disability visibility in reproductive health care. We seek to bring awareness to the daily lived experience of those with disabilities, and recognize that provision of comprehensive and safe reproductive health care is an act of personal autonomy and self-determination. Briefly summarizing the history of reproductive rights in the context of disability, this review will also offer guidance on improving access, quality, and delivery of inclusive and patient-centered care.

The scope will focus on contraception and abortion as it relates to disability. However, we recognize that gender-affirming care, preconception counseling, prenatal care, and preventive screenings (including screening for sexually transmitted infections, cervical cancer, and intimate partner violence) are equally essential elements of comprehensive sexual and reproductive health care.

## Reflexivity statement

The authors would like to acknowledge that they represent a collective of cisgender individuals of various sexualities contributing to the experiences of living with and without various disabilities.^13^ Three of the four are health care providers to reproductive-aged patients with disabilities of various backgrounds and identities. These positionalities likely influence the recommendations of this article. The authors also acknowledge that the concise language used to describe and define individuals with disabilities does not fully reflect the vast diversity of this community nor the lived experiences of all. The authors also acknowledge that the word Queer, reclaimed in this review as an inclusive term referring to those with sexual orientations and gender identities that are not exclusively heterosexual or cisgender, has a complicated history and that this perception is not universally accepted.

Finally, the authors would like to acknowledge the greater disability community. According to the World Health Organization’s 2011 World Report on Disability,^14^ over 16%–19% of the world’s population are estimated to be living with disability. Although beyond the scope of this review, the authors appreciate the global scope of disability and multi-dimensional, lived experiences among diverse individuals with disabilities encountering vast societal, structural, and systems-based barriers when accessing health care.^14^ We will focus primarily on the intersection of disability and reproductive rights within the context of the American legal and cultural systems.

## Ableism and the history of reproductive oppression

Current estimates suggest that at least 12% of American reproductive-aged women identify as having one or more disabilities^15^ and an estimated 16% of individuals worldwide have a disability.^1^ Though individuals with disabilities have equal rights to comprehensive sexual and reproductive health care, barriers to equal access persist. Social inequities and health disparities encountered by individuals with disabilities are not biological but are due rather to structural and societal barriers imposed upon these individuals.^15^ Implicit to these barriers are the intersecting concepts of ableism, “the systems of social power that devalue the bodies and lives of individuals with disabilities,” and stratified reproduction, “the preferential valuing of ‘white fertility of higher socioeconomic status over that of poor women of color’” as defined by Wu et al. (2019).^15^ The interaction of ableism and stratified reproduction “devalues the fertility of individuals with disabilities not only on perceived ability but also other intersecting identities, including race, class, and sexuality.”^15^

In the United States, the intersection of disability rights and reproductive justice is complicated. The medical establishment has been one of the most salient perpetrators of the disability community through a long-standing and sordid history of reproductive coercion, forced sterilization, institutionalization, guardianship, sexual violence, discrimination, and bias since the 19th century.^16^ This history of reproductive violence disproportionately impacts disabled people of color and individuals with other intersecting identities.^3^ Acknowledging and reconciling this history and the innumerable injustices imposed on the disability community is critical to improving access to comprehensive reproductive care and achieving reproductive justice.

## Forced and non-consensual sterilization

Forced sterilization is inextricably linked to the American Eugenics Movement of the late 19th and early 20th centuries as an effort to limit reproduction based on eugenics, racism, and ableism.^3,16,17^ In the 1927 landmark case *Buck v. Bell*, the US Supreme Court^18^ upheld the power of the state to forcibly sterilize individuals with disabilities. Throughout the early 20th century, almost 70,000 individuals were forcibly sterilized, disproportionately affecting individuals with disabilities, Queer, Black, and Indigenous communities.^16^ By the 1970s, forced or non-consensual sterilizations were commonly funded by medical, state, and federal institutions within the United States.^3,16,19,20^ These state-sanctioned and federally funded sterilizations oversaw the forced sterilization of more than 100,000–150,000 Black, Indigenous communities, people of color, and incarcerated individuals.^21^ Ultimately, the use of federal dollars for forced sterilization was prohibited in the 1973 US Supreme Court^22^ decision on *Relf v. Weinberger*, a case that established standards for informed consent.^20^ Despite the significance of these cases, *Buck v. Bell* has never been overturned, and today, forced sterilization remains legal at the federal level.^3^ Currently, 31 states and the District of Columbia allow forced sterilization, with its explicit prohibition in only three states.^3,21^

Forced sterilization is one of many manifestations of systemic discrimination and structural racism imposed on individuals with disabilities and multi-marginalized communities. Simultaneous early efforts to distribute birth control by prominent family planning activists such as Margaret Sanger were also fueled by racist and eugenic ideology.^23^ Today, these systems of reproductive oppression manifest in discriminatory clinical practice, legislative restrictions, inaccessibility, and institutionalism.

## The intersection of disability rights and reproductive justice

Disability rights are a critical reproductive issue within reproductive justice, yet these movements have often been considered in opposition. SisterSong, the largest national multi-ethnic reproductive justice collective in the United States, defines reproductive justice as the right to

have their bodily integrity, privacy, and personal autonomy respected; freely define their own sexuality, including sexual orientation and gender identity and expression; decide whether and when to be sexually active; choose their sexual partners; have safe sexual experiences; decide whether, when, and whom to marry; decide whether, when, and by what means to have a child or children, have access over their lifetimes to the information, resources, services, and support necessary to achieve all the above, free from discrimination, coercion, exploitation, and violence.^2,24,25^

Both disability rights and reproductive justice are human-rights frameworks centering on autonomy, dignity, and justice; yet their alignment has been historically complicated by misinformation, miscommunication, and exclusivity, especially surrounding abortion.^3,26^ This history of sexual violence, structural racism, ableism, and stratified reproduction have built discriminatory systems that perpetuate barriers to exercising bodily autonomy, achieving reproductive equity, and accessing comprehensive reproductive health care.^3^ Likewise, the fight for reproductive rights and the legal right to abortion has historically occurred in environments “dense with misinformation and stigma about the prenatal diagnosis of disability.”^3^ For many Americans, having an abortion is deeply personal. Moreover, the use of disability as a rationale for or against abortion is inappropriate and fails to recognize the nuance and complexity of abortion.^24^

Existing collaborations by the leadership of Queer, Black, and Indigenous activists and liberators are reframing the history and conversations to be more inclusive, safe, and representative of multi-marginalized individuals and communities.^3^ Strengthening the alliance between disability and reproductive justice activists coordinates efforts to ensure disability inclusion in reproductive spaces. Likewise, aligning the Disability and Reproductive Justice Movements fosters collaboration, upholds patients’ right to contraception and abortion, and supports the greater goal of access to comprehensive reproductive health care.^26^

In the following, we will discuss specific barriers to contraception and abortion care as it relates to individuals with disabilities. We must better understand these barriers to health care for individuals with disabilities in order to restore justice after enduring historical atrocities and create space and opportunities for equitable access to comprehensive reproductive health care.

## Mechanisms of inequity and barriers to health care

Individuals with disabilities have the same sexual and reproductive health needs as any other large, diverse population, yet substantial barriers to accessing health care services remain.^15^ Disability theory asserts that health inequities related to disability are created by structural barriers that render the world inaccessible and are not a result of the disabilities themselves.^15^ This review will specifically focus on barriers to reproductive health care. Economic insecurity, coverage for family planning services, lack of accessible health care facilities, provider discrimination/knowledge, guardianship, and policies that perpetuate ableism are a few of the many barriers imposed on the disability community.^15^ We recognize that people with disabilities living at the intersection of other marginalized identities encounter compounding barriers to health care.^27^ Identifying and addressing these barriers is critical to improving access.

## Financial barriers

Variations in economic security exist for people with disabilities and may greatly affect their ability to access health care services. People with disabilities experience a disproportionate exclusion from the workforce^28^ as only 25% of individuals with disabilities are employed.^28^ Of those who are employed, a median wage gap of $12,000 exists, meaning individuals with disabilities make 0.66 to every dollar compared to their non-disabled counterparts.^28^ Thus, individuals with one or more disabilities are twice as likely to live below the poverty level and are twice as likely to report unmet health needs due to financial barriers.^27,28^ Attaining economic security is further complicated by the economic burden of rising health care costs within an expensive health care system.^28^ Compared to the 11.4% of non-disabled people who were uninsured, 8.5% of individuals with a disability still lack health insurance according to the 2021 US Census Bureau Health Insurance Coverage report, the most recent year for which data is available.^29^

Access to contraception and abortion is equally paramount to maintaining economic security. Individuals living in states with comprehensive reproductive health care and access to contraception have demonstrated a reduced probability of living in poverty.^28^ Passage of the Patient Protection and Affordable Care Act in 2011 significantly improved patients’ ability to access and afford contraception. Under this act, preventive services, including Food and Drug Administration (FDA) approved contraception, are provided to patients without cost-sharing, guaranteeing coverage for all US FDA-approved methods.^27^ However, employer-imposed religious and moral exemptions to providing contraception have since restricted universal access to this coverage.^27,28^ Examples of this restriction are illustrated by *Hobby Lobby Stores, Inc. v. Sebelius* in 2012 and *Burwell v. Hobby Lobby Stores* in 2014, in which the US Supreme Court denied Hobby Lobby and Mardel Incorporated’s request for an injunction against employers’ health care plan contraceptive requirement, which was later reversed by the Court of Appeals holding that corporations were protected under the Free Exercise Clause of the First Amendment.^30^

Likewise, many people with disabilities are insured through Medicaid or Medicare.^27^ A 2013 report from the Guttmacher Institute on sexual and reproductive health services suggests one in four women of reproductive age who are Medicaid enrollees obtain contraception through Medicaid.^12,31^ Similarly in 2016, 38% of individuals with disabilities were insured by Medicaid programs.^27^ While Medicaid programs require coverage of family planning services, state-specific variations in coverage and patient qualification impose additional barriers. Restrictions such as the Hyde Amendment prohibit coverage for abortion under Medicaid, disproportionately affecting people with disabilities.^2^ Likewise, other legislative actions such as Executive Order 13535 in 2010 have reinforced a commitment to the preservation of the Hyde Amendment’s restriction of federal funds for abortion.^32^ Furthermore, Medicare plans are not required to cover contraceptive services.^27^

People with disabilities who are also low-income, underinsured, or uninsured face an equivalent challenge when accessing Title X clinics for care. The Title X Family Planning Program is the only federally funded family planning program, including major provisions for clinical care, professional training, research, public education, and information.^12^ Created in 1970 to provide affordable contraception, family planning, and preventive services, its clinics previously served more than four million family planning users in 2016.^12,33,34^ However, the Title X gag rule passed in 2019 and reversed in 2021 prohibited Title X providers from referring patients for abortion and blocked Planned Parenthood health centers from receiving Title X funding.^34^ This rule decreased available providers and health centers by 25%, with over 30 states losing some or all Title X resources, affecting over 19 million persons of reproductive age by widening contraception and abortion deserts.^27,31,33^ While the Biden-Harris administration ended the Title X gag rule in 2021, service sites previously forced out of the Title X network have decreased the available capacity by 46%;^12^ likewise, the Title X Family Planning Program has received no increase in funding in the 2023 fiscal year since 2014.^12^

## Inaccessible medical facilities

Variations in mobility, the use of a variety of assistive devices/technology, and/or variations in communication may exist for people with disabilities. Facility compliance and prioritization of external and internal accommodations vary greatly and affect patients’ ability to access these clinics and medical care facilities. Likewise, absence of accessible medical equipment contributes to patient apprehension and delay in seeking care, dissatisfaction, safety, and quality of care provided.^35^

The passage of the ADA in 1990 mandated equal access to health care services and ushered changes in the built environment as an effort to make public spaces more accessible.^36^ Subsequent passage of the ADA Standards for Accessible Design in 2010 requires facility compliance with enforceable, accessibility standards.^36^ Specific modifications such as curb cuts, pool lifts, and paratransit options were instituted to remove architectural barriers; yet, these standards do not ensure accessibility for the wide range of accommodations that individuals with disabilities may require.^36^ Lack of appropriate accommodations, such as inaccessible medical equipment including height-adjustable examination tables to enable self-transfer, availability of trained and assisted staff, and communication accommodations for visual or hearing-impaired patients (or with visual, hearing limitations) inhibit care and worsen patient apprehension.^37,38^

Even with appropriate accommodations, lack of accessible transportation is an additional barrier obstructing reliable access to reproductive care in individuals who may rely on outside sources for transportation.^35^ Difficulty scheduling care around these constraints, ensuring accessible referrals, inflexible office procedures, and multi-process workups further obstruct patient access to health care.^35,39^

## Provider discrimination and competency

Significant deficits in the content and funding of inclusive, culturally competent, and evidence-based sexual education exist and vary dramatically between states.^2^ Only 30 states in the United States and the District of Columbia require sexual education, with only six states and the District of Columbia providing alternative resources for accessible curriculum and only three states explicitly including people with disabilities in mandated education requirements.^2,10^ This results in a dearth of comprehensive sexual education on subjects such as consent, sexual identity, sexual orientation, and sexual health for people with disabilities.^2,10^ Furthermore, lack of sexual education decreases the ability to identify consensual scenarios, explore appropriate relationships, and increase the risk of sexual assault and intimate partner violence.^2,10,28^

People with disabilities are more likely to experience discrimination due to clinicians’ assumptions regarding reproductive health care needs, ignorance of their lived experience, and sexuality.^28^ Many providers, social workers, and other human service professionals neglect sexual health needs or interests due to a pervasive assumption that individuals with disabilities do not have sex.^24^ A pervasive notion that people with disabilities are unable to consent further obstructs their ability to assert their sexuality and self-advocate.^2^ Some clinicians report discomfort or ill-preparedness to provide comprehensive or sufficient information to patients with disabilities about gynecologic health, sexual health, contraception, and abortion and fail to engage in patient-centered discussions and care.^10,28,40,41^ Health care providers’ negative stereotypes surrounding sexuality, intimacy, and parenthood regarding the disability community not only perpetuate biases but also contribute to disproportionate contraceptive method prescribing patterns and increased incidence of surgical sterilization.^1,28,42^

## Guardianship

Some individuals with disabilities may not be viewed as competent to decide their own health care, and are placed into court-appointed guardianship to assist with medical decision-making.^27^ These guardians can be parents, siblings, spouses, or strangers who act as professional guardians depending on jurisdiction and individual circumstances.^27^ Legally appointed guardians are given the power of substituted decision-making, and harmful stereotypes or false beliefs surrounding disability may impact how guardians make decisions for persons in their care, resulting in coercive contraceptive counseling or access denial.^27,28^ In addition, people under guardianship may not feel comfortable disclosing personal health information.^27^ Because guardians may also have access to medical health records, people under guardianship lack privacy and may not feel safe to have candid discussions about their health care needs.^27^ Thus, several states are working to provide alternatives to guardianship, such as supported decision-making, a concept that would allow people with disabilities to choose who will help them make decisions.^3^ In supported decision-making models, individuals with disabilities retain their primary decision-making capacity by selecting supporters, such as family members, friends, and professionals, to help them understand, consider, and communicate.^19^ This gives individuals with disabilities the tools to make their own informed decisions.^19^

## Improving comprehensive reproductive health care

Every patient, regardless of ability, should have access to the educational and communication tools necessary to express and achieve their sexual and reproductive health goals. Access to contraception and abortion is a matter of bodily autonomy and is a critical reproductive right.^27^ Access to contraception and abortion also maintains economic security, promotes healthy sexual expression, and affords the ability to plan pregnancy if desired.^27^ Despite this, the reproductive health care needs of people with disabilities are often significantly neglected. Patients engaging in coitus with anatomy necessary for pregnancy who do not wish to become pregnant should be counseled about the possibility of pregnancy. Likewise, concerns surrounding vulnerability to non-consensual scenarios and unintended pregnancy contribute to the worries of many caregivers.^4^ Open-ended questions and patient-centered communication should be utilized such that patients are their own prime decision makers, with providers and caregivers providing a supporting role.^43^ As experts in their health and values, patients should be empowered with each interaction.^43^ Likewise, sufficient time for a clinic visit should be provided to allow for the patient to express themselves using any necessary communication accommodations.^24,39^

## Menstrual health

For some patients and caregivers, menstruation may be bothersome or challenging, particularly if irregular or heavy.^4^ If desired, methods for menstrual management and/or contraception, if needed, should be discussed and offered. Clinicians may anticipate requests/interest around menstrual suppression and management and should be able to offer information and guidance to both the patient and, if applicable, the caregiver using communication styles that match the literacy needs/level of the patient and caregiver.^4^ Requests for menstrual suppression not directly communicated by the patient should be considered critically: Is menstruation affecting the quality of life or is this a preference by the caretaker? When considering menstrual suppression as a request from a caretaker or guardian when a patient is unable to express their preference, such requests must be balanced with the patient’s expressed desires and best interests.

People with disabilities maintain equal rights to the full range of management options as their non-disabled peers.^44^ Clinicians are responsible for referring to the US Medical Eligibility Criteria to review the desired contraceptive method with the patient’s medical history and safety category for use. Likewise, clinicians should engage in open-ended conversations to understand any accessibility or mobility preferences or requirements when discussing method use. The specific needs and preferences of each individual patient should always guide the choice of the best method.^10^

Health care systems, medical practices, and clinicians should strive to accommodate a diversity of patient needs. For pelvic examinations and long-acting reversible contraceptive procedures, including implant and intrauterine device placement, providers should be educated on accommodating techniques, including alternative examination positions beyond dorsal lithotomy.^39^ Open-ended questions to understand individual accommodation needs or preferences should be used, deferring to the language they use to describe their needs.^39^ Providers should always ask for permission and direction prior to assisting the patient, regardless of the patient’s apparent ability to participate in any interaction.^39^ Prior to the examination, providers should ensure the patient’s consent to all individuals present in the room.^39^ Trauma-informed care should always be performed during the pelvic examination, and/or any procedure, including long-acting reversible contraception placement, which should also be offered under anesthesia if preferred.

## Contraception

People with disabilities share a similar age of menarche and menstrual patterns compared broadly to those without,^4^ and are equally likely to be sexually active and to experience pregnancy as people outside of the disability community.^15,45,46^ Despite this, people with disabilities experience decreased rates of contraception use, disproportionate method use, decreased contraceptive education, and higher risk of STI.^47^ Notably, 83% of women report never being asked at initial or subsequent appointments about contraception.^40^ Disparities in contraception access are due to financial barriers, inaccessibility, non-inclusive or culturally incompetent care, guardianship, and/or religious refusal.^24,27^ Consequently, these disparities in contraceptive access increase the risk of sexual assault and the rate of unintentional pregnancy, and worsens maternal morbidity and mortality in pregnancy.^3,16,19,20^

Provider discrimination within the historical context of racism and ableism has resulted in disproportionate method use: either provision of no specific method or provision of permanent sterilization among individuals with disabilities.^16,27^ Many providers have historically denied requests for specific contraception from individuals with disabilities due to personal biases or assumptions of diminished sexual activity.^27^ Previous research indicates that individuals with disabilities are less likely to be prescribed long-acting reversible contraception or oral contraceptive pills.^27,42,45^ Instead, patients are more likely to be offered no contraception or surgical sterilization as a method of contraception.^15,27,42^ These discrepancies in contraception utilization are compounded for individuals at the intersection of other multi-marginalized identities or communities.^3^ The 2011–2015 National Survey of Family Growth found that 41% of Black, disabled women used sterilization as contraception, compared to 28% of White, disabled women.^3^ Clinicians should critically assess their prescribing patterns to ensure practices are unbiased, evidence-based, and patient-centered. It is not unreasonable to provide sterilization to adults with disabilities who seek this service; however, it is not equitable to either provide or deny sterilization purely on the basis of disability status alone. Likewise, the provision of contraception purely because of disability, regardless of method, may also be coercive, as all individuals have the right to choose or not to choose to contracept.

The American College of Obstetricians and Gynecologists asserts that caregivers may request a hysterectomy for definitive amenorrhea and contraception in individuals with disabilities.^4^ Hysterectomy for cessation of menses and/or tubal ligation for permanent contraception may be considered only after alternatives have been trialed and thoroughly exhausted.^4^ Requests for a hysterectomy and/or tubal ligation not directly communicated by the patient should be considered critically: Are menses significantly affecting the patient’s quality of life despite adequate evaluation and treatment options? Is parenthood or the capacity to become pregnant significantly bothersome to the patient or is this a preference of the caretaker? An understanding of the irreversible nature of surgical procedures and their significant morbidity and mortality, when compared to other non-invasive methods of contraception/menstrual management, is critical.^4^ Especially in light of the US history of forced sterilization and reproductive oppression, all efforts must be made to promote the patient’s autonomy when considering a hysterectomy. Protective regulations for Medicaid-funded sterilization exist to prevent procedural coercion, such as a 30-day waiting period between the time of consent and sterilization and the use of a standardized consent form.^4,48^ Providers should confirm individual state’s laws regarding sterilization and hysterectomies as consent, legal procedure, and process may vary from state to state.^4^ However, in light of safe and effective options for contraceptive and menstrual management, the risks and costs of surgical sterilization may be disproportionate to their benefit.

Provision of comprehensive reproductive health care is an exercise of reproductive autonomy and a matter of reproductive justice. Having the ability, resources, and education to make informed or supported decisions regarding menstrual health and contraception is critical and cannot be fully realized without accounting for and responding to the needs of the disability community.^24^ This requires an understanding of the varied experiences of persons with disabilities, their support systems, and their desires.

## Abortion

Comprehensive access to abortion is necessary to support an equitable society, empower reproductive decision-making, maintain economic security, and express bodily autonomy and self-determination.^5,3,28,49,50^ Abortion is a matter of health equity. Individuals who are denied abortions are more likely to remain in relationships where interpersonal violence is present, and are more likely to experience serious pregnancy complications, including eclampsia and death.^28^

Despite a long-standing history of reproductive oppression and lack of bodily autonomy imposed by the medico-legal establishment, disability has largely been overlooked or excluded in discussions about abortion rights. The Reproductive Justice Movement has not always centered on the specific challenges encountered by people with disabilities or considered how these histories and experiences add nuance and complexity to the issues of reproductive autonomy.^28^ Though the topic of disability-selective abortion may complicate the relationship between the Reproductive and Disability Justice Movements, there have been key moments of combined efforts from the Reproductive and Disability Justice Movements.^3,50^ Since 2019, an upswing in proposed bills that forbid abortions on the basis of a fetal diagnosis of disability have also surfaced, otherwise known as “selective abortion bans.”^51^ Examples such as North Carolina’s House Bill 453 utilize disability as a rhetorical device to restrict access to abortion, rather than protecting people with disabilities.^52^ As stated by disability advocate Rebecca Cokley, “removing our right to control our bodies [and] our personhood, has been common practice by the ‘well-meaning’ nondisabled public for centuries.”^52^

The overturning of *Roe v. Wade* and the subsequent wave of abortion bans has grave implications for individuals with disabilities.^53^ As of January 2023, abortion is banned in 12 US states, and over 29% of the total US population seeking abortions are living in states where abortion is unavailable or severely restricted.^52^ Abortion restrictions deepen the profound inequities to access long endured by Queer, Black, and Indigenous communities, low-income communities, people of color, and people with disabilities.^52^ States with the greatest restriction to abortion access are also noted to have the highest rates of disability and chronic illness. While pregnancy poses serious health in general, those with chronic conditions and underlying health issues are at even greater risk for complications.^53^ Furthermore, this population of individuals may rely on medications that are contraindicated during pregnancy.^28^ Restricting or removing the option for pregnancy termination for people with disabilities can be a matter of survival. Abortion bans’ threat to bodily autonomy also exacerbates the existing limitations on the health and overall well-being of disabled people.^28,53^ Moreover, failures of the medical establishment and economic system to provide Black, Indigenous communities, and people of color with equal access to safe and affordable access to health care compound the detrimental effect of abortion restrictions.^52^

For people with disabilities, other barriers to abortion care exist. These may be financial or logistical barriers, such as a lack of accessible transportation, which can make it hard to travel regionally, or difficulty scheduling appointments around transportation constraints.^28^ According to a 2022 study by the Guttmacher Institute, patients in restricted states were more likely to pay out of pocket for care (87% vs 42%), rely on financial assistance (22% vs 11%), and indicate difficulty paying for abortion (54% vs 28%).^54^ Many people with disabilities may rely on faith-based providers for assistance with transportation, personal care, and making medical appointments.^28^ Providers may impose personal refusal on patients seeking an abortion to deny assistance with abortion-related care or referral. Conversely, the criminalization of individuals seeking or providing abortion care has widened disparities in abortion access.^28,52^ Combined with targeted restrictions of abortion providers and facilities, funding restrictions, mandatory waiting periods, parental involvement, state-mandated counseling, and gestational age limits devastate access to care or put abortion care entirely out of reach.^28^

Reducing the compounding barriers imposed on the disability community is imperative to improving abortion access. Full access to safe and effective options for abortion is central to protecting individuals’ lives and protecting reproductive rights and equity.

## Limitations

The authors recognize that while this review attempts to be thorough and culturally competent, it is beyond its scope to fully address the multi-faceted problems that this population and other international communities may face when seeking reproductive health care. It additionally acknowledges the subconscious biases that the authors’ lived experiences present while constructing this review and recognizes that this article may not accurately reflect the lived experience of every person with disability. Finally, it acknowledges that the included statistics were last updated at the time of publication and likely do not convey the most up-to-date information on abortion restrictions.

## Conclusion

Access to comprehensive and inclusive reproductive health care is essential, regardless of disability status. Despite the hard-fought successes of activists, leaders within the Disability and Reproductive Justice Movements, and policies such as the Americans with Disabilities Act, barriers to accessing equitable care remain.

Individuals with disabilities have endured a long history of reproductive oppression, ableism, stigmatization, and exclusion from being centered and empowered within their own conversations about contraception and abortion access. Understanding barriers and discriminatory structures is critical to challenging ableism and achieving access to comprehensive, inclusive reproductive health care. This article acknowledges the importance of including providers, scholars, legislators, and administrators with disabilities in the creation of further research, policies, and laws surrounding disability rights, to the degree they are willing to be present and reflexive in these roles. Disability visibility and promotion within reproductive spaces are critical and would benefit from policies and research driven in part by lived experience. Queer, Black, and Indigenous communities have already played a significant role in framing discourse around reproductive rights as inclusive of marginalized communities.^3^ Amplifying diverse voices within the disability community is necessary to identify policy solutions that best center the needs of those with the greatest barriers.^3,28^ Finally, reconciling reproductive oppression and discriminatory systems against individuals with a disability requires intentional, interdisciplinary partnerships within and beyond medicine. Using a collaborative framework between the Disability Rights and Reproductive Justice Movements, there are concrete actions that we can take:

1. Providers must focus on the foundational importance of communication. We should engage in open-ended, shared decision-making about contraception and abortion, acknowledging that people with disabilities are the experts of their own health and bodies. Providers should ensure adequate time for each clinical encounter and uphold evidence-based practices when providing contraception and abortion care.
2. Scholars must explore the intersection of disability and reproductive justice communities and frameworks to better understand how societal structures hinder comprehensive reproductive health care access. Collaboration is paramount for centering disability, diverse identities, and historically marginalized identities to achieve reproductive justice and representation for all. Scholars should also seek to provide a greater understanding of the collective implications of the *Dobbs v. Jackson Women’s Health Organization* decision on the disability community.
3. Medical systems must increase clinic accessibility by advocating for accessible equipment and information, assistive technologies, and communication accommodations in health care facilities.^5^ Clinics and health care systems should prioritize and provide training of medical professionals and staff on accessibility issues and equitable care for persons with disabilities in the clinical setting.^5,39^
4. Medical providers and institutions must address ableism in the field of medicine and their own practice, assert that individuals with disabilities are whole, sexual beings, and critically assess for discrimination in individual clinical practice and health care delivery. Providers have a critical responsibility to recognize personal bias and to combat stereotypes, prejudices, and harmful clinical practices relating to persons with disabilities, especially those regarding sexual and reproductive health.
5. State and regional legislatures must promote and advocate for the passage of legislation that centers disability and reproductive health care, expands access and protections to funding family planning services, and prioritizes mandated, evidence-based, and inclusive sexual education accessible to all communities. All available measures should be taken to modify or abolish existing discriminatory laws, regulations, and practices against people with disabilities, and prioritize centering the voices of multi-marginalized identities. Legislatures must also immediately end the continued permission of forced sterilization. Finally, legislatures, policy makers, community allies, and reproductive and disability justice advocates must continue to advocate for unrestricted, comprehensive access to abortion.

## Declarations

**Ethics approval and consent to participate:** Not applicable.

**Consent for publication:** Not applicable.

**Availability of data and materials:** Not applicable.
